# Analysis of M2 macrophage-associated risk score signature in pancreatic cancer TME landscape and immunotherapy

**DOI:** 10.3389/fmolb.2023.1184708

**Published:** 2023-07-04

**Authors:** Dashuai Yang, Fangrui Zhao, Yang Su, Yu Zhou, Jie Shen, Kailiang Zhao, Youming Ding

**Affiliations:** ^1^ Department of Hepatobiliary Surgery, Renmin Hospital of Wuhan University, Wuhan, China; ^2^ Department of Oncology, Renmin Hospital of Wuhan University, Wuhan, China; ^3^ Department of Gastrointestinal Surgery, Tongji Hospital, Tongji Medical College in Huazhong University of Science and Technology, Wuhan, Hubei, China

**Keywords:** M2 macrophages, pancreatic cancer, WGCNA, prognostic model, immunotherapy

## Abstract

**Background**: M2 macrophages perform an influential role in the progression of pancreatic cancer. This study is dedicated to explore the value of M2 macrophage-related genes in the treatment and prognosis of pancreatic cancer.

**Methods**: RNA-Seq and clinical information were downloaded from TCGA, GEO and ICGC databases. The pancreatic cancer tumour microenvironment was revealed using the CIBERSORT algorithm. Weighted gene co-expression network analysis (WGCNA) was used to detect M2 macrophage-associated gene modules. Univariate Cox regression, Least absolute shrinkage and selection operator (LASSO) regression analysis and multivariate Cox regression were applied to develop the prognostic model. The modelling and validation cohorts were divided into high-risk and low-risk groups according to the median risk score. The nomogram predicting survival was constructed based on risk scores. Correlations between risk scores and tumour mutational load, clinical variables, immune checkpoint blockade, and immune cells were further explored. Finally, potential associations between different risk models and chemotherapeutic agent efficacy were predicted.

**Results**: The intersection of the WGCNA results from the TCGA and GEO data screened for 317 M2 macrophage-associated genes. Nine genes were identified by multivariate COX regression analysis and applied to the construction of risk models. The results of GSEA analysis revealed that most of these genes were related to signaling, cytokine receptor interaction and immunodeficiency pathways. The high and low risk groups were closely associated with tumour mutational burden, immune checkpoint blockade related genes, and immune cells. The maximum inhibitory concentrations of metformin, paclitaxel, and rufatinib lapatinib were significantly differences on the two risk groups.

**Conclusion**: WGCNA-based analysis of M2 macrophage-associated genes can help predict the prognosis of pancreatic cancer patients and may provide new options for immunotherapy of pancreatic cancer.

## Background

Pancreatic cancer is one of the worst prognoses of all malignant parenchymal tumours-the 5-year survival rate is only around 9% (Bray et al., 2018; Christenson et al., 2020). Age has been identified as a risk factor for pancreatic cancer. With the global trend of ageing, the incidence of pancreatic cancer is increasing every year (Siegel et al., 2018; Siegel et al., 2020; Huang and Setiawan, 2022). In recent years, advances have been made in the comprehensive treatment of pancreatic cancer, such as immune checkpoint blockade therapy, which has provided new treatment options for patients with chemotherapy-resistant pancreatic cancer (Le et al., 2015; Ullman et al., 2022). However, immunotherapy still requires large randomised prospective studies to confirm its role in improving the prognosis of patients with pancreatic cancer (Fan et al., 2020; Ostios-Garcia et al., 2021; Ullman et al., 2022).

The tumour microenvironment is the internal environment upon which tumour cell genesis, growth and metastasis depend (Yang et al., 2021). The tumour microenvironment in pancreatic cancer consists of a large number of tissue interstitial, immune cell infiltrates and other components, of which tumour-associated macrophages are the main component. M2-type macrophages are the main type of tumour-associated macrophages, which play an irreplaceable role in functions such as trophic competition, inflammatory response, metabolic changes, tumour metastasis and immunosuppression (Feig et al., 2012; Noy and Pollard, 2014; Cohen et al., 2015; Davies and Taylor, 2015).

However, few existing studies have investigated the potential role of tumour-associated macrophages in the tumour microenvironment of pancreatic cancer as a mechanism for chemoresistance and immunotherapy in pancreatic cancer (Ip et al., 2017). Consequently, this study was based on the construction of co-expression networks through The Cancer Genome Atlas (TCGA) and Gene Expression Omnibus (GEO) and International Cancer Genome Consortium (ICGC) databases employing a WGCNA analysis approach to identify prognostic models of macrophage-associated genes in the pancreatic cancer microenvironment. This study systematically investigated the potential mechanisms of the genes in the model and the response of patients in different risk groups to chemotherapy and immunotherapy to provide a practical reference model for individualised clinical treatment of pancreatic cancer.

## Data downloading and processing

RNA sequencing (RNA-seq) data, clinical profiles and tumour mutation burden data for pancreatic cancer patients were obtained from the Cancer Genome Atlas data (https://portal.gdc.cancer.gov/repository). Meanwhile, clinical profiles and RNA expression data for pancreatic cancer patients from the GSE71729 database were downloaded from the Gene Expression Omnibus (GEO) repository (nlm.nih.gov/gds/). Gene expression data and prognostic data from the International Cancer Genome Consortium (ICGC) database of pancreatic cancer patients were utilized for model validation (https://dcc.icgc.org/projects/LIRI-JP). Inclusion criteria (Bray et al., 2018): survival time >0 and (Christenson et al., 2020) complete clinical information.

### Evaluation of immune cell infiltration

Immune cells in the tumour microenvironment affect tumour progression and treatment efficacy. CIBERSORT predicts the proportion of 22 immune cells in tumour sample expression data based on linear support vector regression principles. Based on results at *p* < 0.05, the proportion of immune cells in patients with pancreatic cancer samples from the TCGA and GEO databases was calculated and the results presented by the ggplot2 R package.

### WGCNA

Genes associated with M2 macrophages in pancreatic cancer are identified by an algorithm using weighted gene co-expression network analysis (WGCNA), which is a common analytical method for exploring the relationship between gene sets and the phenotype of interest. The R-based “WGCNA” package was built for co-expression networks of genes in TCGA and GEO, respectively. A proximity matrix was constructed based on the best soft threshold β from 1–20 to match the gene distribution to a connection-based scale-free network. Neighbourhood relationships were then converted into a topological overlap matrix (TOM) and clustered in a chain hierarchy based on the mean of different TOM-based metrics. Similar genes are introduced into the same candidate modules using a “dynamic tree cutting” algorithm. Correlations between the module signature genes and the phenotypes of interest were analysed using Pearson’s correlation test (*p* < 0.05). Finally, the expression of genes in the co-expression modules of WGCNA was performed to correlate the proportion of immune cell infiltration in patients.

### Building the model

The results of the WGCNA analysis of the TCGA and GEO databases were used to select the set of genes most relevant to M2 macrophages in the module and to take the intersecting genes of both. The intersecting genes were first integrated with patient survival data from TCGA; then univariate COX regression was used to identify the genes that affected patient survival. Next a penalty function was generated using lasso regression to compress the coefficients of the variables to prevent overfitting of the model. Finally, the results of the multifactorial COX regression analysis were confirmed for M2 macrophage-related genes affecting survival in pancreatic cancer patients.

Risk score = βgene A × expr gene A + βgene B × expr gene B+…+ βgene N × expr gene N, expr is the mRNA expression of the pivotal gene and β is the corresponding regression coefficient in multivariate genetic Cox regression analysis.

### Model validation

Results based on TCGA multifactorial COX regression analysis were screened from the ICGC database for the appropriate genes and combined with survival times to validate the data from the modelling group. Time-dependent ROC curves were employed to validate the accuracy of risk scores in predicting patient prognosis.

### Nomogram

The nomogram provided a visual representation of a patient’s prognosis. Based on the risk score and the patient’s clinical data a nomogram was constructed to predict the patient’s prognosis at 1 year, 2 years and 3 years. Calibration curves (by bootstrap method with 500 resamples) and receiver operating characteristic (ROC) curves were applied to evaluate the nomogram.

### Sample tumour mutation burden analysis

The TCGA database provides the raw tumour mutation burden data for the samples. The study first downloaded the original tumour mutation burden for each sample of pancreatic cancer samples and calculated the value of the tumour mutation burden for each sample. Waterfall plots for the high-risk and low-risk groups were plotted by “maftools”. In addition, survival curves were plotted between the four subgroups based on the median mutational load of pancreatic cancer patients.

### GSEA enrichment analysis

To explore the ranking of genes in the model that lie in the correlation of different phenotypes, functional annotations were explored utilising the c2. cp.kegg.v7.4. symbol and c5. go.v7.4. symbol collections against the Gene Set Enrichment Analysis (GSEA) software. The first six of the annotated results were selected for display and defined as statistically significant with a two-sided *p*-value of <0.05.

### GSVA enrichment analysis

To explore the pathways by which genes in the M2 macrophage-associated model may influence the pancreatic cancer tumour microenvironment. The MSigDB database (https://www.gsea-msigdb.org/gsea/msigdb) was used for pathway analysis of M2 macrophage-associated genes.

### The relationship between risk models and the tumour microenvironment

To further explore the relationship between the role of M2 macrophage-associated risk models in the immune microenvironment, XCELL, timer, quantitative, MCPcer, EPIC, Sibe sorting and Sibe sort-abs were employed to explore the relationship between risk scores and patient immune function. Scores for each sample were first assessed based on gene expression using the ESTIMATE algorithm. Secondly, Spearman correlation analysis was applied to evaluate the relationship between risk scores and tumour immune function.

#### Immunological target prediction

Immunotherapeutic targets play a decisive role in immunotherapy and immune tolerance. Expression of M2 macrophage-associated genes and 47 and immunotherapeutic targets between high and low risk groups were systematically analysed. The immune round of cancer cells determines the efficacy of immunotherapy. The immune panel score (IPS) is an important measure of the immune prototype. The immune score of a sample was integrated by calculating the scores for antigen presentation, effector, suppressor and checkpoint separately.

### Drug sensitivity prediction

M2 macrophage-associated models may influence the effectiveness of chemotherapy in patients. Differences in drug sensitivity between high- and low-risk groups were explored based on the “pRRophetic” “ggplot2”. Differences in half-maximal inhibitory concentrations (half-inhibitory concentrations) of various chemotherapeutic agents were evaluated between high- and low-risk groups of patients with pancreatic cancer using the Wilcoxon signed-rank test.

### Real-time PCR

20 Total RNA from pancreatic cancer tissue and paired paracancer tissue samples was treated with an RNA separator total RNA extraction reagent (Vazyme). The cDNA was synthesized from total RNA using NovoScript^®^ plus an all-in-one first strand cDNA synthesis kit (Novo protein). GAPDH was appllied as an internal control. Primers used for RT-PCR assay are shown in **Additional File 1: Table 1**.

### Statistical analysis

The Wilcoxon rank sum test was used as a backup to compare differences between two groups. kruskalWallis test was used to compare differences between three groups and more. Kaplan-Meier method and log-rank test were used for prognostic analysis. All data analysis was done by R (4.1.2, https://www.r-project.org/) software. Bilateral *p* < 0.05 was considered statistically significant.

## Results

### Patient data

After collating and screening the clinical and expression data of the patients, 172 samples from the TCGA database, 79 from the GEO database GSE71729 and 80 from the ICGC database were included in the study. The median follow-up time for patients with pancreatic cancer in the TCGA, GEO and ICGC databases were 15.61 [interquartile range (IQR): 8.98–22.49] months, 13 (IQR: 6.00–22.00) months, 15.20 (IQR: 8.66–26.46) months respectively.

### Tumour microenvironment analysis

**FIGURE 1:**
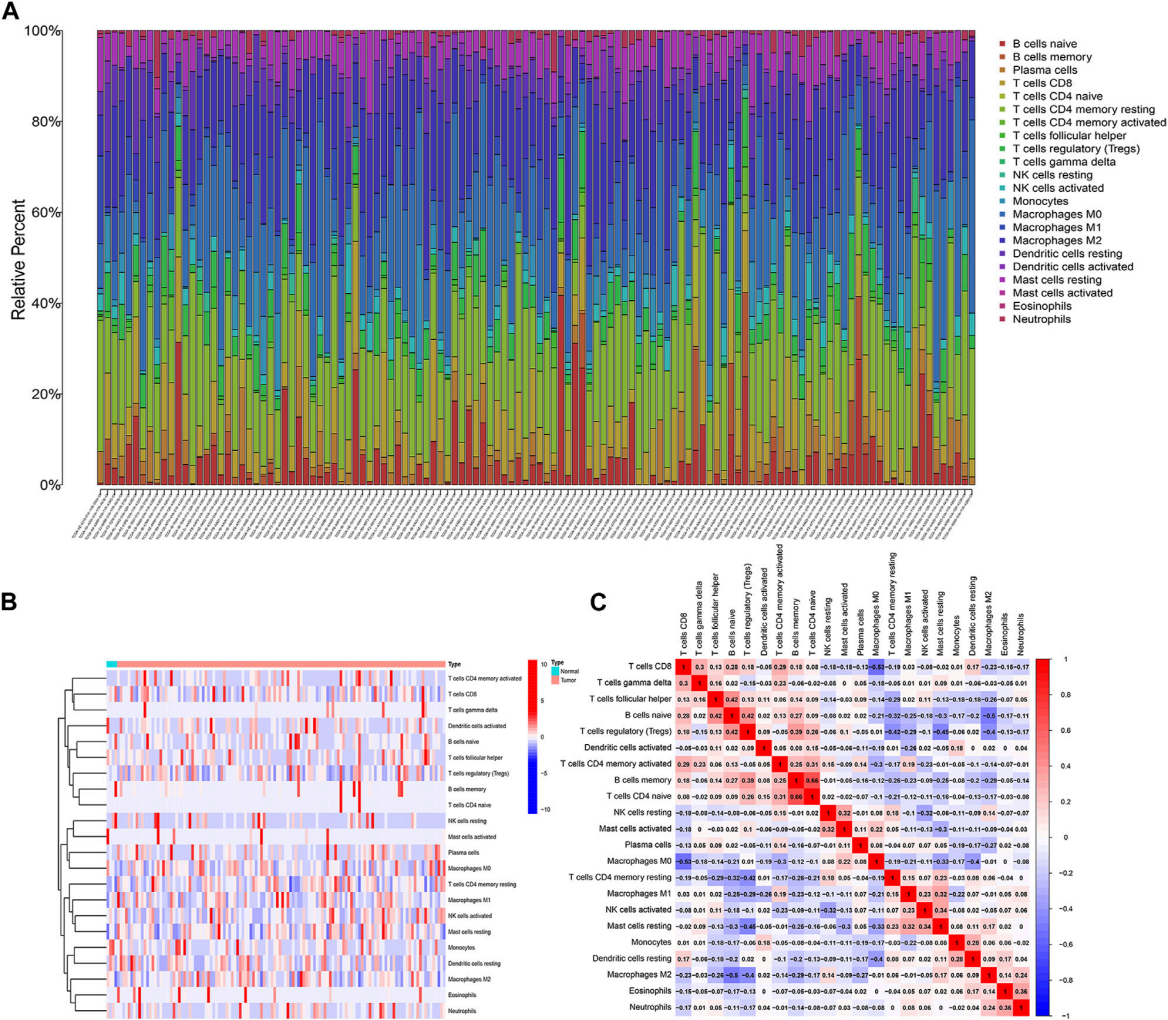
Analysis of immune infiltration in pancreatic cancer. **(A)** 22 immune cell subpopulation in TCGA pancreatic cancer samples. **(B)** Proportional heat map of 22 immune cell in TCGA pancreatic cancer samples. **(C)** Correlation analysis of 22 immune cells.

The proportions of 22 immune cells were calculated for each sample of pancreatic cancer patients in the TCGA and GEO databases were calculated based on the CIBERSORT algorithm, respectively (Additional File 1: Supplementary Tables S2, S3). As shown in Figure 1A, the row names represent each sample and the different colours of the cylindrical plot represent the proportion of different immune cells. The heat map (Figure 1B) demonstrates the difference in immune infiltration between normal and tumour tissue. The correlation heat map suggests a potential relationship between the 22 immune cells (Figure 1C).

## WGCNA

**FIGURE 2:**
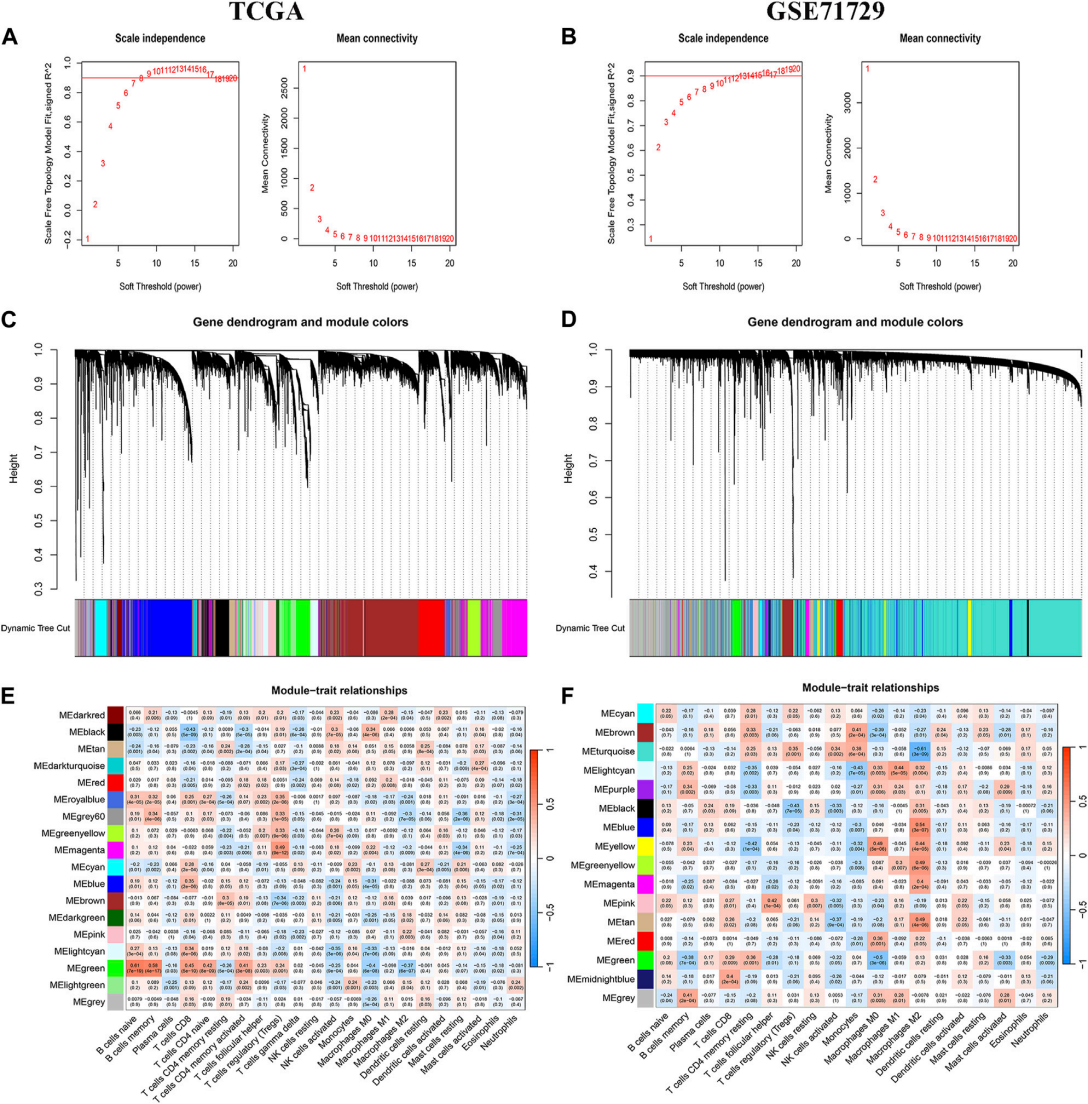
WGCNA Analysis. **(A, C, E)** TCGA database aggregation of gene modules with similar expression models based on the WGNCA algorithm and correlation analysis of modules with immune infiltrating cells. **(B, D, F)** GEO database aggregation of gene modules with similar expression models based on the WGNCA algorithm and correlation analysis of modules with immune infiltrating cells.

A WGCNA co-expression network was built based on gene expression files (TCGA: 19,819 genes, GEO: 19,014 genes) and immune cell infiltration results. The optimal soft threshold power (TCGA: b = 8,GEO: b = 11) when the scale-free topology index first reached 0.9 was set as the first set of power values to build the scale-free network **(** Figures 2A, B). Genes with similar expression patterns were grouped into the same gene module to form a hierarchical clustering tree based on a “dynamic tree cutting” algorithm (module size = 60). Finally, a weighted hierarchical clustering analysis was performed to obtain the clustered gene modules (Figures 2C, D). The highest correlations with M2 macrophages in the TCGA and GEO databases were green and turquoise respectively. The intersecting genes of the two modules were finally identified as the set of M2 macrophage-associated genes for the next analysis(Figures 2E, F).

### Building the model

**FIGURE 3:**
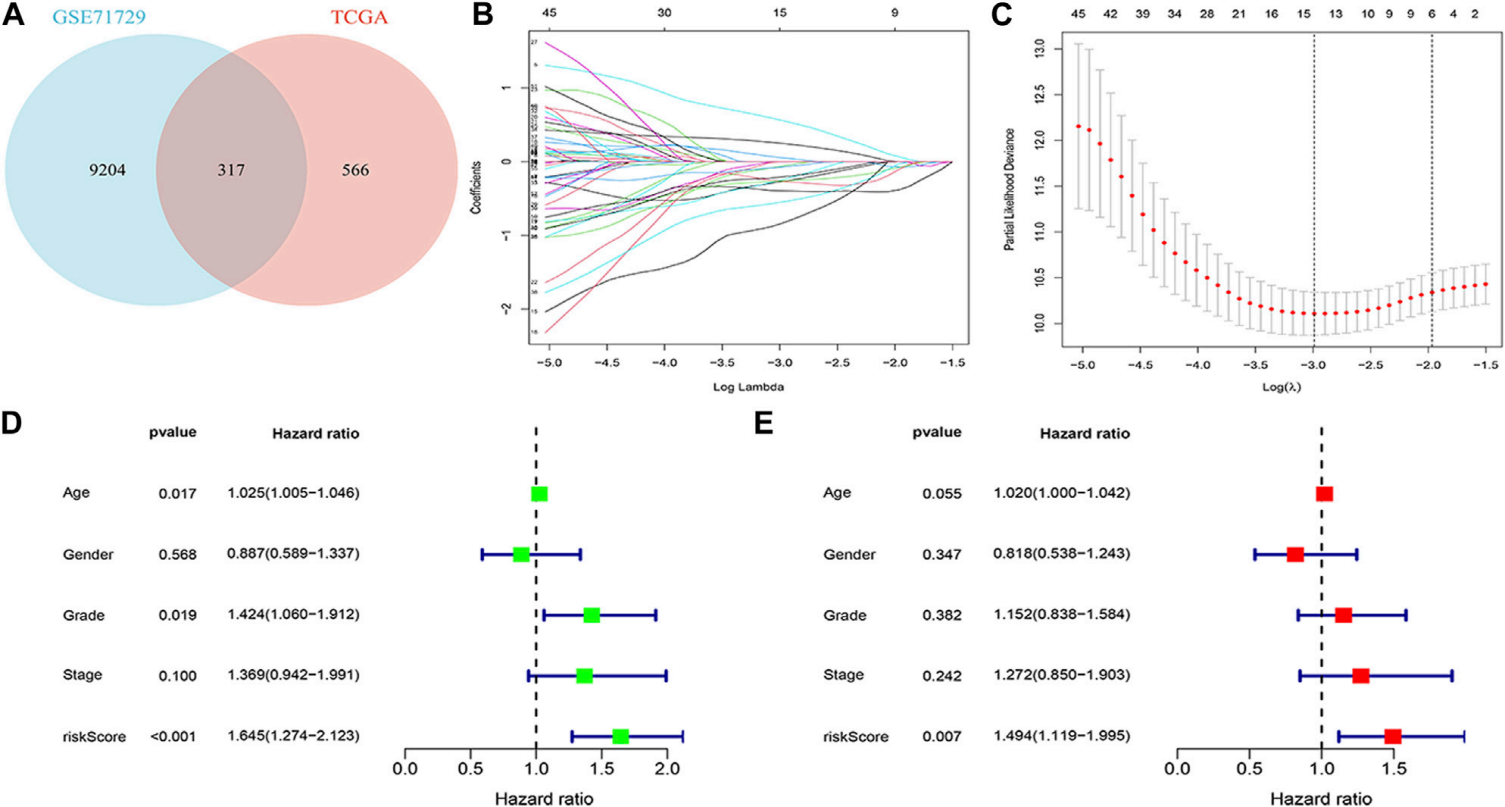
Building a risk model **(A)** Venn diagram. **(B)** Log (λ) change curves of regression coefficients. **(C)** Tenfold cross-validation of adjusted parameter choices in lasso regression. Vertical lines are plotted from the best data according to the minimum criterion and 1 standard error criterion. **(D, E)** Results of univariate and multivariate COX regression analyses.

The 317 genes in the TCGA and GEO databases were finally recognised as M2 macrophage-associated genes (Figure 3A). Clinical data and follow-up information of patients were extracted from the TCGA database and merged with the expression of the 317 genes. Sixty genes were screened for association with patient prognosis after univariate COX regression analysis. The results of the Lasso regression were used in the multifactorial COX regression analysis (Figures 3B, C), and the final 9 genes (ABCB4, APOBEC3C, ENPP6, FGFBP2, LIPE, MT2A, OXER1, PLD4, ZNF589) were selected for model construction (Additional File 1: Supplementary Tables S4, S5). Risk scores were calculated for each sample using the risk score formula and the samples were divided into low and high risk groups depending on the median score. Risk score is an independent prognostic element for patients (Figures 3D, E). Protein expression levels in pancreatic cancer patients were explored based on the HPA database. The results suggested that the protein of the target gene is differentially expressed in normal tissues and pancreatic cancer tissues. Meanwhile, model genes were differentially expressed in both tumor tissues and normal tissues (Additional File 2).

### Validation of the model

**FIGURE 4:**
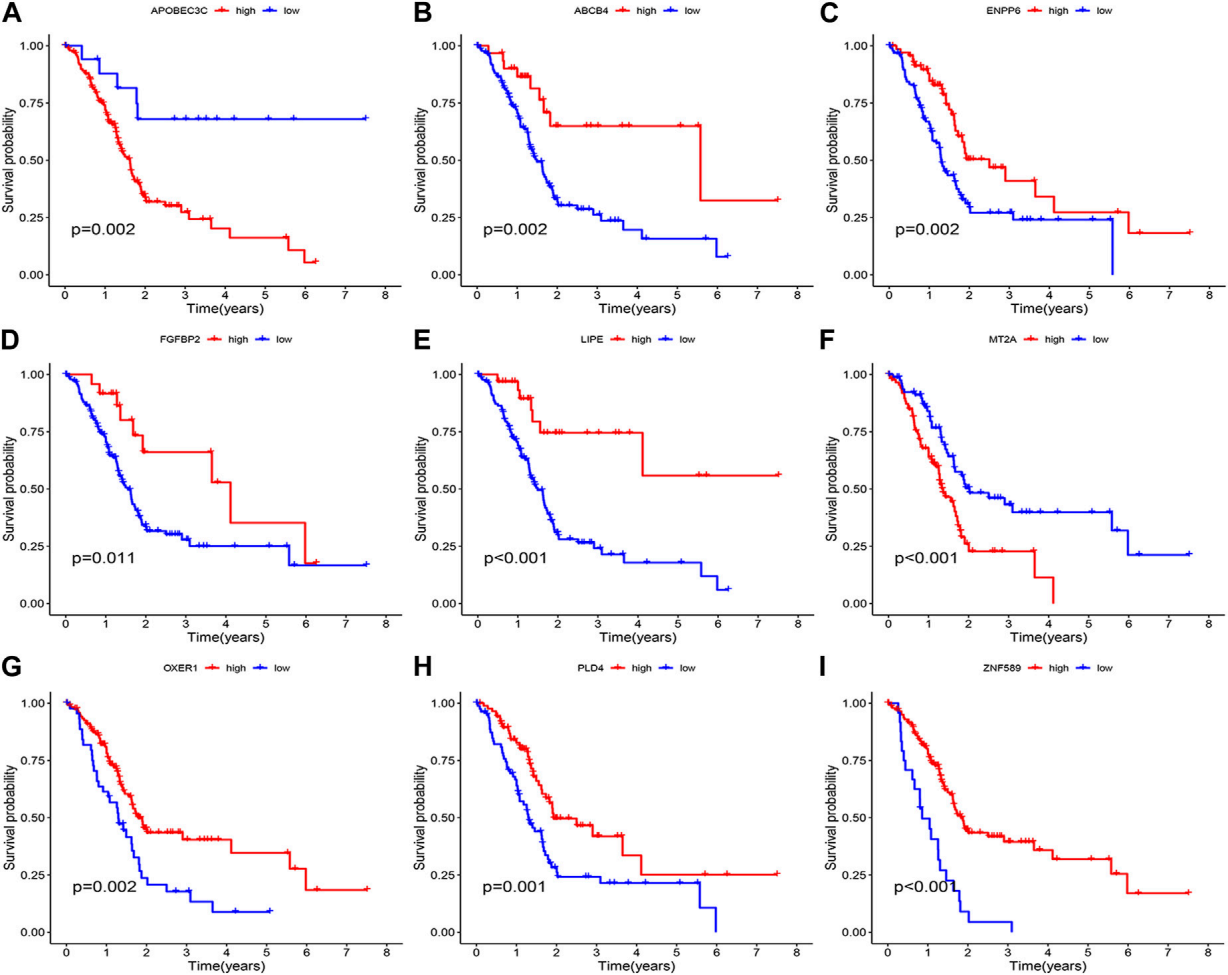
Prognostic analysis of the model gene. **(A)** APOBEC3C. **(B)** ABCB4. **(C)** ENPP6. **(D)** FGFBP2. **(E)** LIPE. **(F)** MT2A. **(G)** OXER1. **(H)** PLD4. **(I)** ZNF589.

**FIGURE 5:**
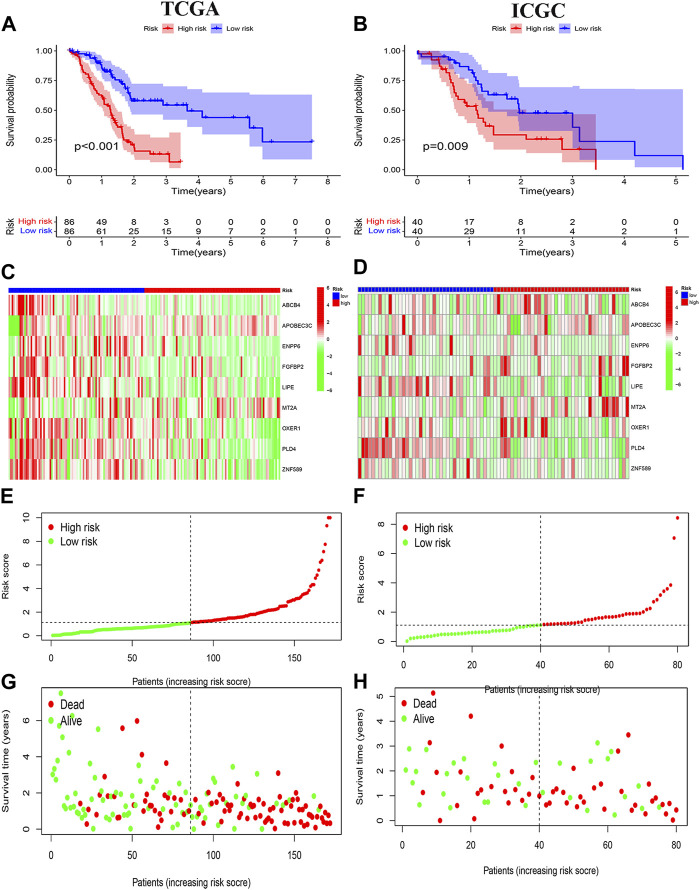
Prognostic analysis. **(A, B)** Risk signature survival analysis in TCGA and ICGC databases. **(C, E, G)** Heat plot, risk score plot and scatter plot based on TCGA dataset. **(D, F, H)** Heat plot, risk score plot and scatter plot based on ICGC dataset.

Survival curves classifying each of the nine genes into high and low risk groups based on median expression levels indicated that the expression levels of all nine genes correlated with patient prognosis (Figure 4). The heat map clearly demonstrated the difference in expression of the model genes between the high- and low-risk groups. Scatterplot of risk scores and patient survival revealed a higher proportion of patients with higher risk scores. The results were validated in the validation group (Figure 5).

### Correlation of clinical variables

**FIGURE 6:**
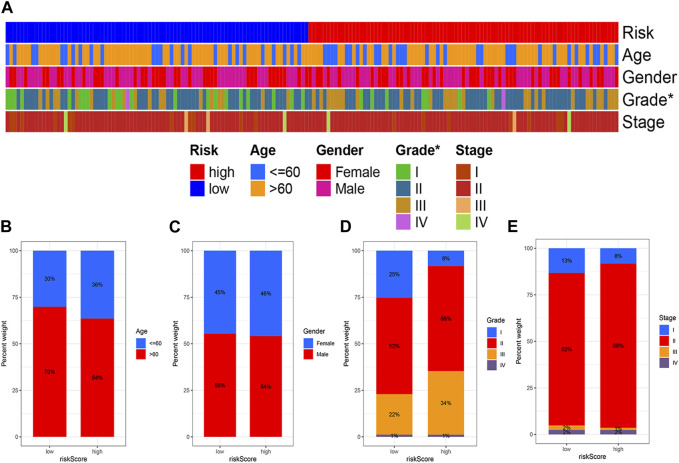
Correlation analysis of risk models and clinical variables. **(A)** Heat map showing clinical characteristics and risk scores for each sample. **(B)** Age. **(C)** Gender. **(D)** Grade. **(E)** Stage. (*p < 0.05; **p < 0.01; ***p < 0.001).

High and low risk groups were correlated with clinical variables. The correlation between high and low risk groups and age, gender, pathological grade and tumour stage, respectively, is shown in Figure 6.

### Predicted prognosis nomogram

**FIGURE 7:**
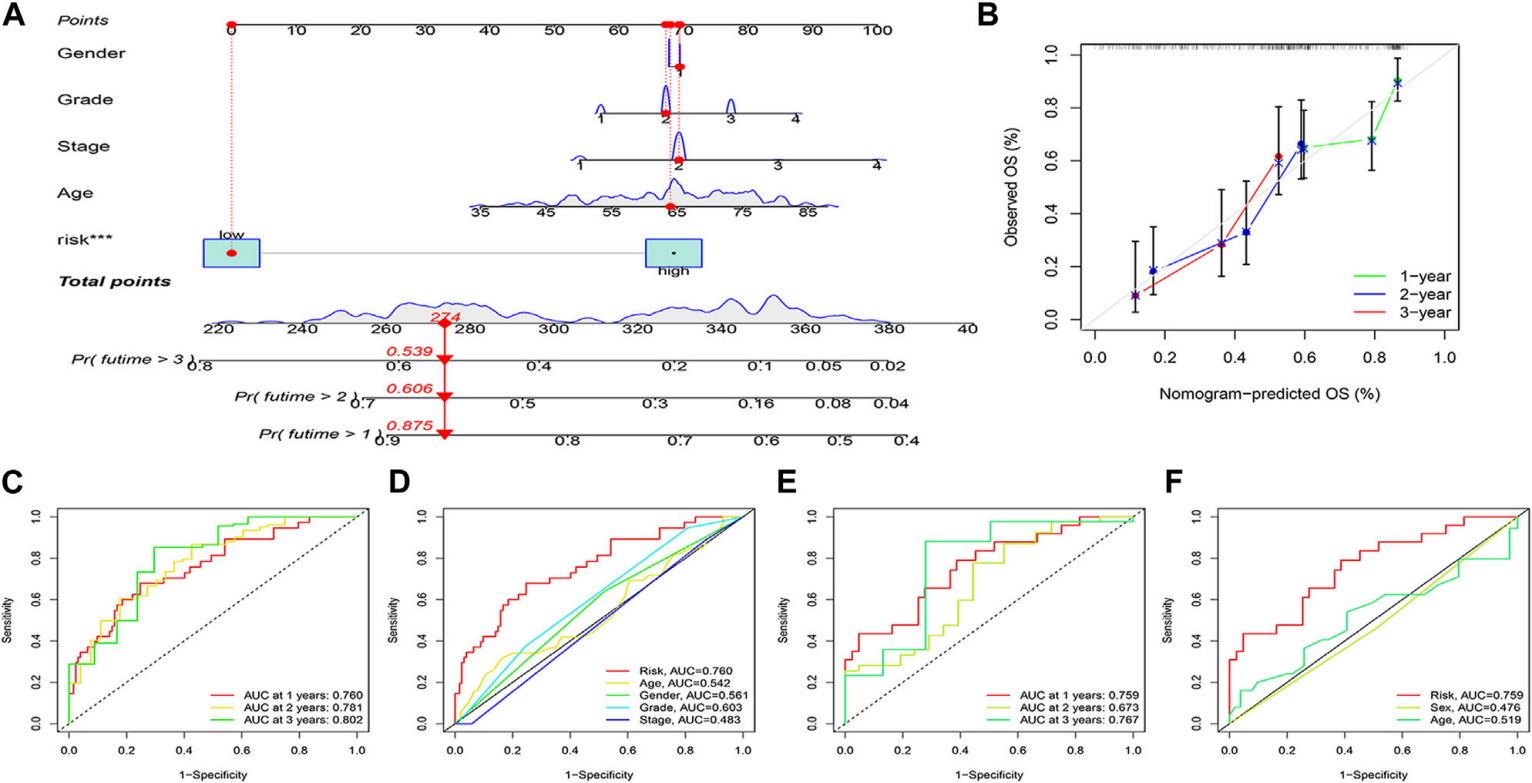
Nomogram and its verification. **(A)** Nomogram of patients’ prognosis at 1–3 years. **(B)** Calibration curves. **(C, D)** ROC analysis based on TCGA dataset. **(E, F)** ROC analysis based on ICGC dataset.

The risk score was combined with clinical information to construct the nomogram predicting the prognosis of patients at 1, 2 and 3 years to enhance the functionality of the risk score in clinical practice. For example, if the total patient score for the example in the nomogram is 274, the overall probability of patient survival at 1, 2 and 3 years is 0.875,0.606 and 0.539 respectively (Figure 7A). Calibration curves showed stable predictive power of the nomogram (Figure 7B). Time dependent ROC curves indicated 1, 2 and 3 years AUC values of 0.760, 0.781 and 0.802 for the modelling group and 0.759, 0.673 and 0.767 for the validation group (Figures 7C, E), indicating that the model has high predictive ability of the model. Simultaneously, the AUC values for risk scores were higher than for other clinical variables (Figures 7D, F).

### GSEA enrichment analysis

**FIGURE 8:**
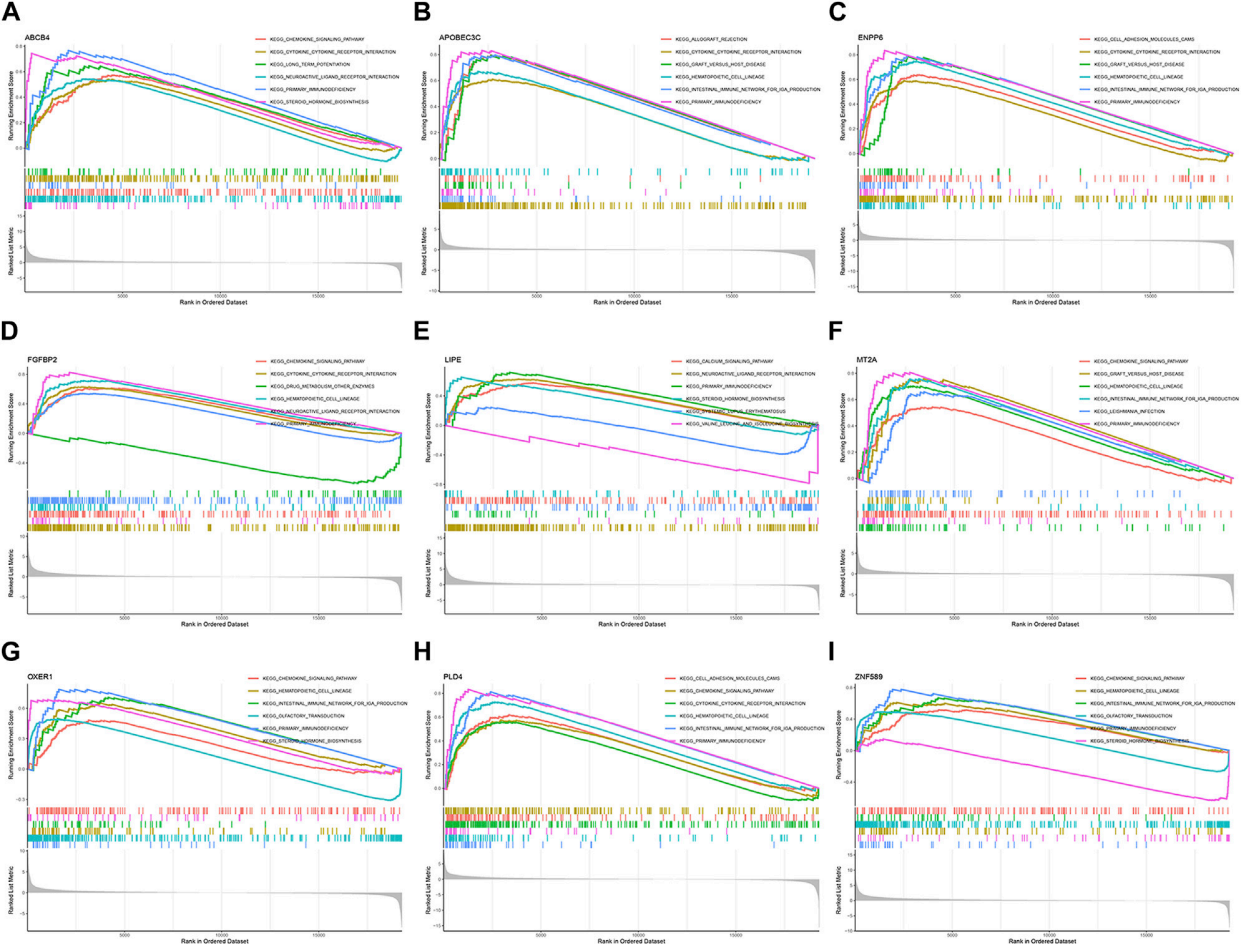
**GSEA** enrichment analysis. **(A)** ABCB4. **(B)** APOBEC3C. **(C)** ENPP6. **(D)** FGFBP2. **(E)** LIPE. **(F)** MT2A. **(G)** OXER1. **(H)** PLD4. **(I)** ZNF589.

GSEA enrichment analysis was performed to explore the possible pathways through which M2 macrophage-associated genes affect the immune microenvironment. The genes were divided into high and low expression groups according to their median expression and the differences in signalling pathways between the two groups were investigated. The KEGG enrichment project indicated that ABCB4 was involved in signalling, cytokine receptor interaction, and cellular value-added signalling pathways, APOBEC3C was linked to immune rejection, cytokine receptor interaction, and gastrointestinal immune signalling pathways, ENPP6 mainly affected cell adhesion, cytokine receptor interaction signalling pathways, FGFBP2 was related to academic signalling, drug metabolism, haematopoietic cell pathways, LIPE affected signaling pathways of calcium signaling, biosynthesis, leucine isoleucine synthesis, MT2A was concerned with signaling pathways of chemical signaling, hematopoietic cell lineage, gastrointestinal immunity, OXER1 was associated with chemical signaling, steroid synthesis signaling pathways, PLD4 was engaged in cell adhesion, chemical signaling, cytokine receptor interaction signaling pathways, ZNF58 impacted signaling pathways of chemical signaling, immunodeficiency, and taste perception (Figure 8).

### Tumour mutation burden analysis

**FIGURE 9:**
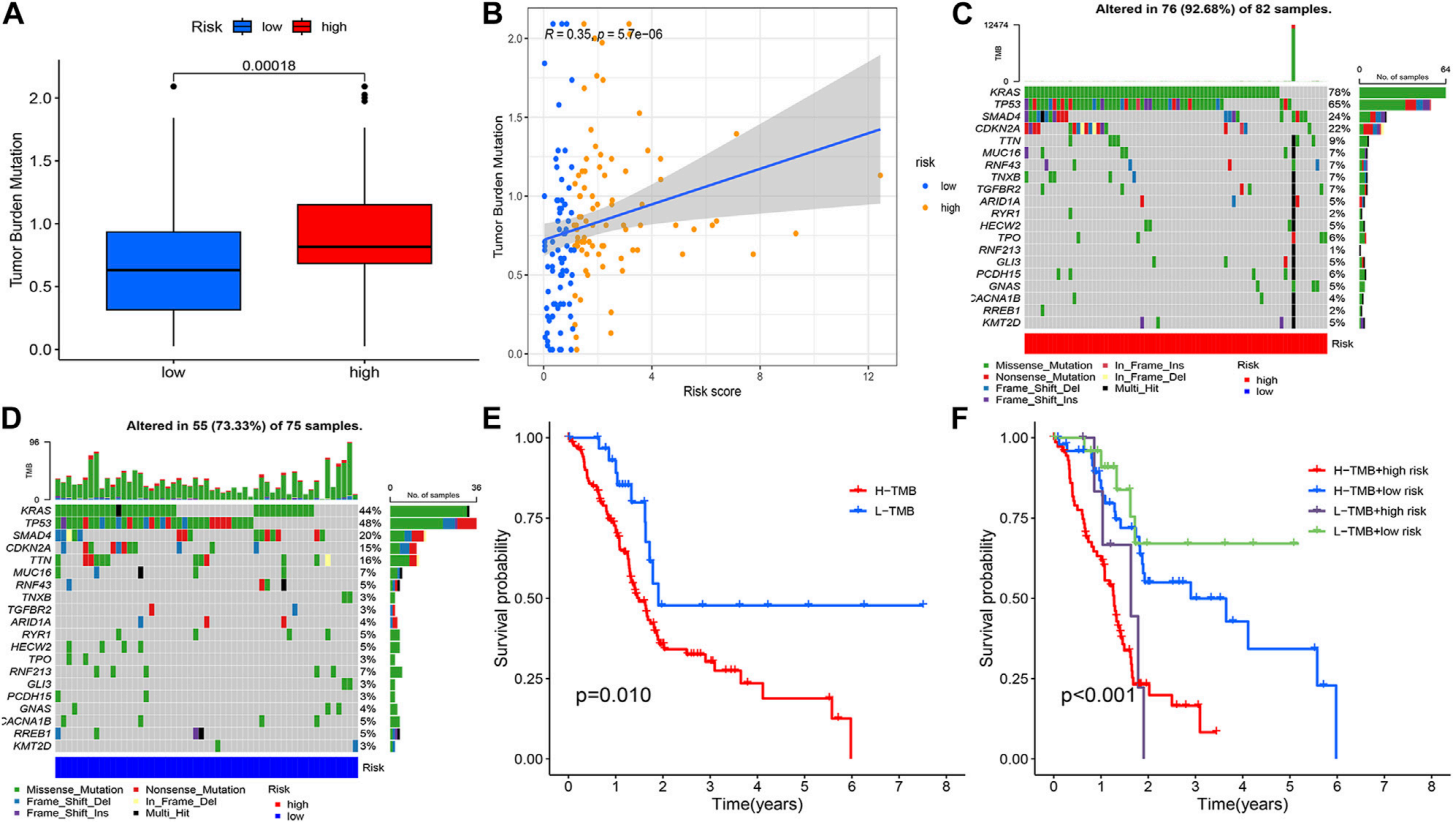
Tumour mutation load analysis, **(A)** Differential counting of tumour mutation burden between high and low risk groups. **(B)** Correlation analysis of risk score and mutation burden. **(C, D)** OncoPrint between high and low risk groups. **(E)** Prognostic analysis of tumour mutation load, **(F)** Prognostic analysis of tumour mutation load in high and low risk groups.

The tumour mutation burden was first calculated for all samples. Statistically significant differences in tumour mutation burden levels between the two groups (Figure 9A). Correlation analysis between risk score and tumour mutation burden indicated that a higher risk score implied a higher tumour mutation burden (Figure 9B). The sample was immediately divided into a high and low tumour mutation group by median tumour mutation value. There was a significant difference in survival between the two groups (*p* < 0.05). Figures 9E, F showed that this finding was validated in the high-risk and low-risk groups.

### The relationship between risk models and the tumour microenvironment

**FIGURE 10:**
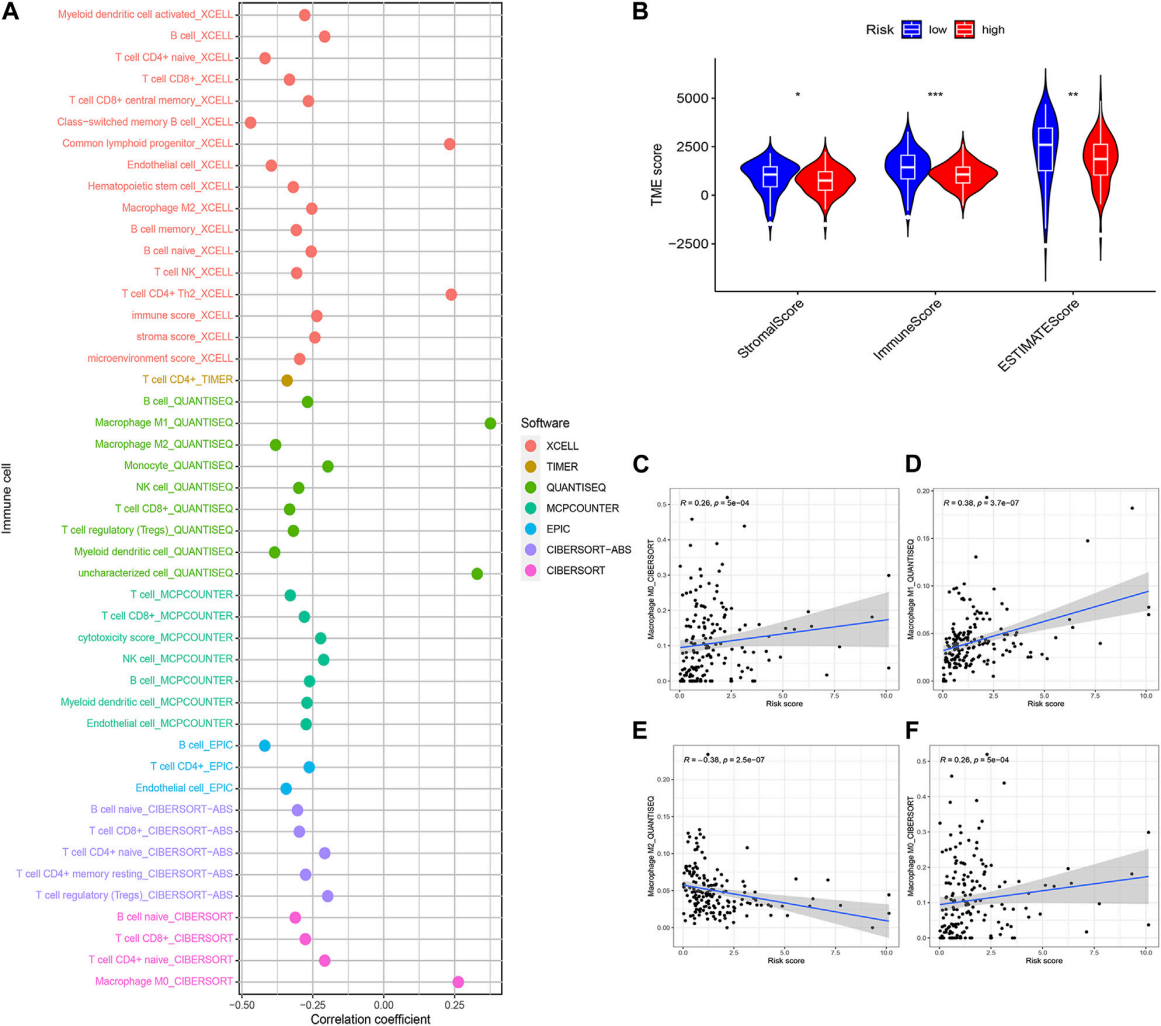
Estimated abundance of tumor-infiltrating cells. Patients in the **(A)** high-risk group had a stronger correlation with tumor-infiltrating immune cells, as shown by the Spearman correlation analysis. **(B)** Association between prognostic risk signatures and central immune checkpoint genes. The asterisks represented the statistical *p*-value. The correlations predicted by the four methods CIBERSORT−ABS **(C)**, CIBERSORT **(D)**, QUANTISEQ **(E)**, and XCELL **(F)** were validated. (*p < 0.05; **p < 0.01; ***p < 0.001).

The potential relationship between model genes and the tumour microenvironment was investigated based on Spearman correlation analysis (Figure 10A). Figure 10B indicated that immune scores, stromal score, and ESTIMATE scores were different between the high-risk and low-risk groups (*p* < 0.05). The results of the correlation between risk scores and tumour microenvironment analysed by the four methods CIBERSORT-ABS (Figure 10C), CIBERSORT (Figure 10D), QUANTISEQ (Figure 10E), and XCELL (Figure 10F) immediately afterwards demonstrated the potential of M2 macrophage-related model genes to influence the pancreatic cancer tumour microenvironment.

### GSVA enrichment analysis

**FIGURE 11:**
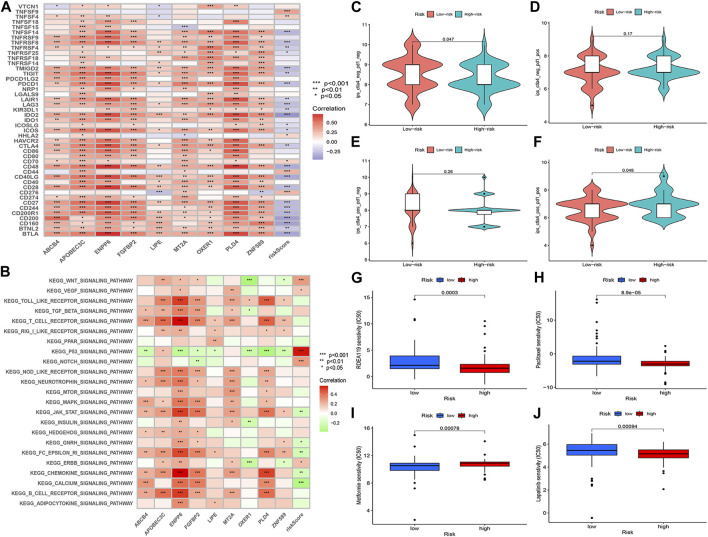
**(A)** GSVA enrichment analysis. **(B)** Correlation analysis of immune checkpoint blockade gene expression levels and risk scores. **(C–F)** IPS score distribution map. Estimates of chemotherapy effect risk scores. **(G)** Metformin. **(H)** Rafatinib. **(I)** Paclitaxel. **(J)** Lapatinib.

GSVA enrichment analysis revealed a negative correlation between ABCB4, ENPP6, FGFBP2, LIPE, OXER1, PLD4, ZNF589 and the p53 signaling pathway. ABCB4, APOBEC3C, ENPP6, FGFBP2, MT2A, PLD4 and the MAPK signaling pathway were positively correlated. ABCB4, ENPP6, PLD4 and the calcium signaling pathway were positively correlated. FGFBP2, PLD4 and calcium signaling pathway were positively correlated (Figure 11A).

### Immunotherapy predictions

The prognostic model for M2 macrophage-associated genes was negatively correlated with most immune checkpoint blockage-associated genes (CD40, IDO2, TNFRSF8, CD48, CD28, PDCD1) and a few immune checkpoint blockage genes (TNFSF9, TNFSF4, CD44, CD276) were positively correlated with the risk score model (Figure 11B). Higher IPS scores in the low-risk group (pd1-negative and ctla4-negative) indicated that the low-risk group was better treated with the new immune checkpoint inhibitors (ICI) (Figures 11C–F). These results demonstrated the potential role of M2 macrophage-related risk groups in predicting the outcome of immunotherapy in patients.

### Predicting chemotherapy drug efficacy

Analysis of the chemotherapeutic drugs’ semi-inhibitory concentrations identified that paclitaxel, rafatinib and lapatinib had a higher drug sensitivity in the low-risk group than in the high-risk group, while metformin had a higher drug sensitivity in the high-risk group. The results of the study showed a correlation between the effect of chemotherapeutic drugs and the prognostic model of M2 macrophage-associated genes (Figures 11G, H).

### Independent sample validation

**FIGURE 12:**
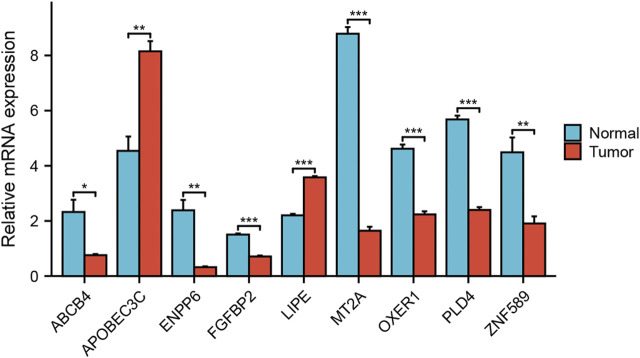
Analysis of expression differences. Verifying the expression of genes that constitute the risk model through RT qPCR.

The gene expression difference was verified by RT-PCR detection of 20 samples of pancreatic cancer patients from Renmin Hospital of Wuhan University. The results showed that APOBEC3C and LIPE were highly expressed in pancreatic cancer tissues. However, ABCB4, ENPP6, FGFBP2, MT2A, OXER1, PLD4 and ZNF589 were low expressed in pancreatic cancer tissues (Figure 12).

## Discussion

Pancreatic cancer is highly aggressive and most patients are diagnosed at an advanced stage and are deprived of effective treatment options (Hidalgo et al., 2015; Karamitopoulou, 2019). The immune system of the body is the last barrier to kill tumour cells. The low immunogenicity and immune escape characteristics of pancreatic cancer reduce the therapeutic efficacy of patients with pancreatic cancer (Liu et al., 2022). Tumour-associated macrophages account for a substantial proportion of the pancreatic cancer tumour microenvironment, and the major part of pancreatic cancer-associated macrophages differentiate into M2-type tumour-associated macrophages (Velasco et al., 2023). Recent studies have demonstrated that M2-type macrophages are involved in immune escape from pancreatic cancer (Campbell et al., 2010; Evan et al., 2022). Therefore, further study to uncover M2-related genes in the tumour microenvironment macrophage-associated genes and the mechanisms of action between the tumour microenvironment may increase new horizons for immune tolerance in pancreatic cancer therapy.

In recent years, there have been some advances in the treatment of solid tumours. For example, immunotherapy has been applied in the treatment protocols for solid tumours such as breast cancer, lung cancer and liver cancer (Link et al., 2018; Locati et al., 2020). However, only a few tumours have achieved favourable clinical outcomes. There is an urgent clinical need for new treatment options to stimulate the patient’s immune system to kill tumour cells. The tumour microenvironment provides a supportive ecological environment for cancer cell development and metastasis. It has been found that in solid tumours macrophages occupy a predominant component of the tumour microenvironment. However, macrophages have a dual role in cancer (Hinshaw and Shevde, 2019; Pittet et al., 2022). In different settings macrophages exhibit different forms of activation. In the classical pathway macrophages differentiate into M1 macrophages in response to stimulation by bacterial products and interferons (Cao et al., 2022). M2 macrophages are produced in the type 2 immune response by factors such as IL-4 and IL-13 via the alternative pathway (Biswas and Mantovani, 2010). The M1 type of macrophage possesses the function of killing tumour cells (Schlundt et al., 2021). In contrast, M2 is involved in the entire process of tumourigenesis and metastasis. It has been shown that m2 macrophages can be recruited by individual tumour initiating cells and thus provided a culture ecology for early tumourigenesis (Gordon and Plüddemann, 2019). Meanwhile, the pro-angiogenic and remodelling matrix of m2 cells can promote tumour growth and metastasis (Raghavan et al., 2021). The immune tolerance that occurs during tumour immunotherapy may be related to the overexpression of suppressive counter-receptors (e.g., PDL1 and PDL2) by m2 macrophages that suppress the body’s immune function (Pushalkar et al., 2018). Therefore, blocking macrophage-associated immunosuppressive targets may be a way to suppress adaptive immune responses. Blocking macrophage-associated immunosuppressive targets may therefore be a potential therapeutic option to suppress adaptive immune responses and enhance the efficacy of immunotherapy (Stakheyeva et al., 2017; Riquelme et al., 2018).

The M2 macrophage-associated genes identified in this study have been reported in the existing pancreatic cancer tumour microenvironment (Mazarico et al., 2016; Saito et al., 2022). Mazarico et al. (2016) discovered that ABCB4 was overexpressed in pancreatic cancer-resistant patients treated with gemcitabine, indicating that ABCB4 may enhance immune escape of tumour cells by affecting macrophage function, leading to resistance to chemotherapeutic agents. Qian (Qian et al., 2020; Qian et al., 2022) revealed that overexpression of APOBEC3C induces genomic instability in pancreatic cancer, increases tumour cell heterogeneity and participates in the remodelling of the tumour immune microenvironment by influencing the function of immune cells. In the tumor microenvironment, ENPP can inhibit the aggregation of immune cells by reducing cGAMP, resulting in enhanced immune escape of tumor cells (Matas-Rico et al., 2021; Borza et al., 2022). Böker (Böker et al., 2022) showed a large number of single nucleotide variants in FGFBP2 in pancreatic tumour cells, and these changes affect the growth and migration of tumour cells. Masi revealed that OXER1 may be involved in the remodelling of the tumour immune microenvironment through multiple pathways and could be a potential target for immunotherapy (Masi et al., 2021). Although LIPE, MT2A, PLD4 and ZNF589 have been studied in other tumours, their relationship with tumour-associated macrophages in pancreatic cancer remains to be investigated. These findings not only provide new insights into the pathogenesis and immune tolerance mechanisms of pancreatic cancer in the future, but also may be potential new therapeutic targets for pancreatic cancer.

Undoubtedly, there are still some limitations in the present study. Firstly, the difference in mRNA expression was verified in tumor and normal tissues. However, further validation in cells and animals should proceed. Secondly, the results of the study need more work before they can be applied clinically.

## Conclusion

The M2 macrophage-associated prognostic model for pancreatic cancer performed excellently in patient prognosis, tumour mutation load analysis, immune checkpoint prediction, and chemotherapy drug sensitivity prediction. Meanwhile, M2 macrophage-related genes may be involved in the targeting of immunotherapy in pancreatic cancer patients.

## Data Availability

The original contributions presented in the study are included in the article/Supplementary Material, further inquiries can be directed to the corresponding authors.

## Abbreviations

RNA-seq, CRNA sequencing; TCGA, The Cancer Genome Atlas; GEO, Gene Expression Omnibus; ICGC, International Cancer Genome Consortium; GSEA, Gene set enrichment analysis; GSVA, Gene set variation analysis; TME, Tumor microenvironment; ICB, Immune checkpoint blockade; WGCNA, Weighted gene coexpression network analysis; TMB, Tumor mutational burden; LASSO, Least absolute shrinkage and selection operator; DCA, Decision curve analysis; KEGG, Kyoto Encyclopedia of Genes and Genomes; ICI, Immune checkpoint inhibitors.
