# Supplementary figures and images for: Analysis of M2 macrophage-associated risk score signature in pancreatic cancer TME landscape and immunotherapy
Source: Front Mol Biosci. 2023 Jul 4;10:1184708. doi: 10.3389/fmolb.2023.1184708 (PMC10352656; doi:10.3389/fmolb.2023.1184708)

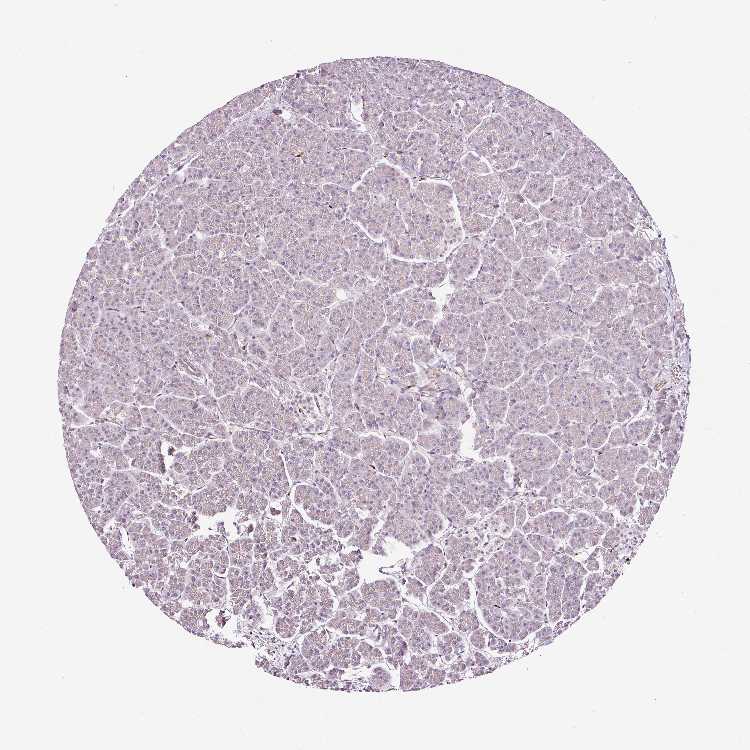

Supplement: Supplementary file 1 [file Presentation1.zip › Supplementary/Additional File 2/ABCB4_Normal.jpg]

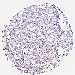

Supplement: Supplementary file 1 [file Presentation1.zip › Supplementary/Additional File 2/ABCB4_Tumour.jpg]

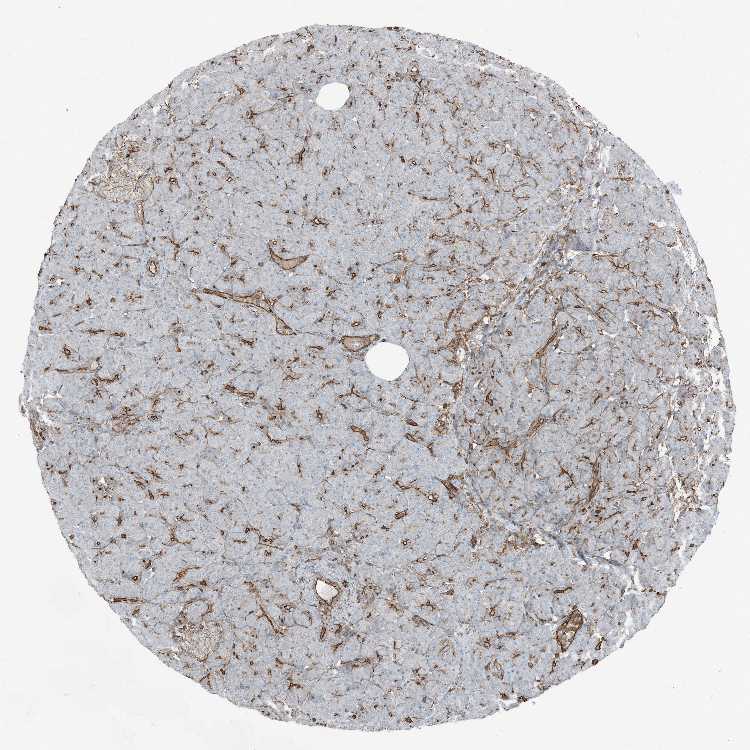

Supplement: Supplementary file 1 [file Presentation1.zip › Supplementary/Additional File 2/APOBEC3C_Normal.jpg]

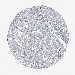

Supplement: Supplementary file 1 [file Presentation1.zip › Supplementary/Additional File 2/APOBEC3C_Tumour.jpg]

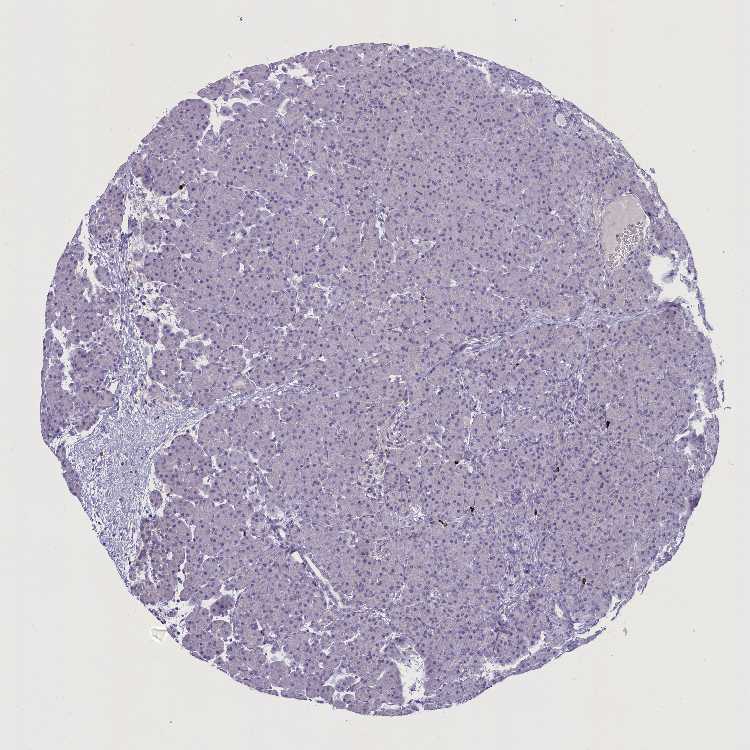

Supplement: Supplementary file 1 [file Presentation1.zip › Supplementary/Additional File 2/ENPP6_Normal.jpg]

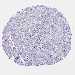

Supplement: Supplementary file 1 [file Presentation1.zip › Supplementary/Additional File 2/ENPP6_Tumour.jpg]

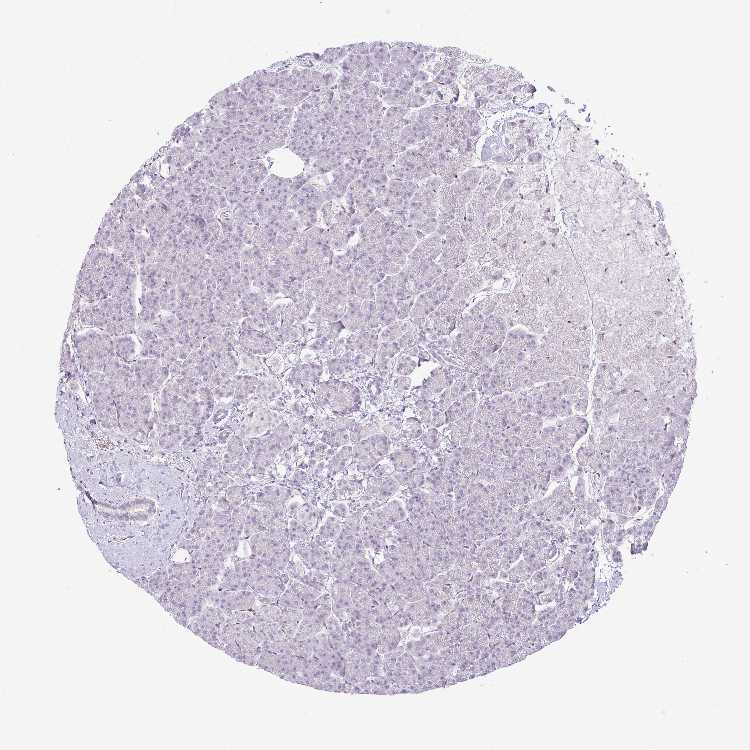

Supplement: Supplementary file 1 [file Presentation1.zip › Supplementary/Additional File 2/LIPE_Normal.jpg]

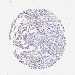

Supplement: Supplementary file 1 [file Presentation1.zip › Supplementary/Additional File 2/LIPE_Tumour.jpg]

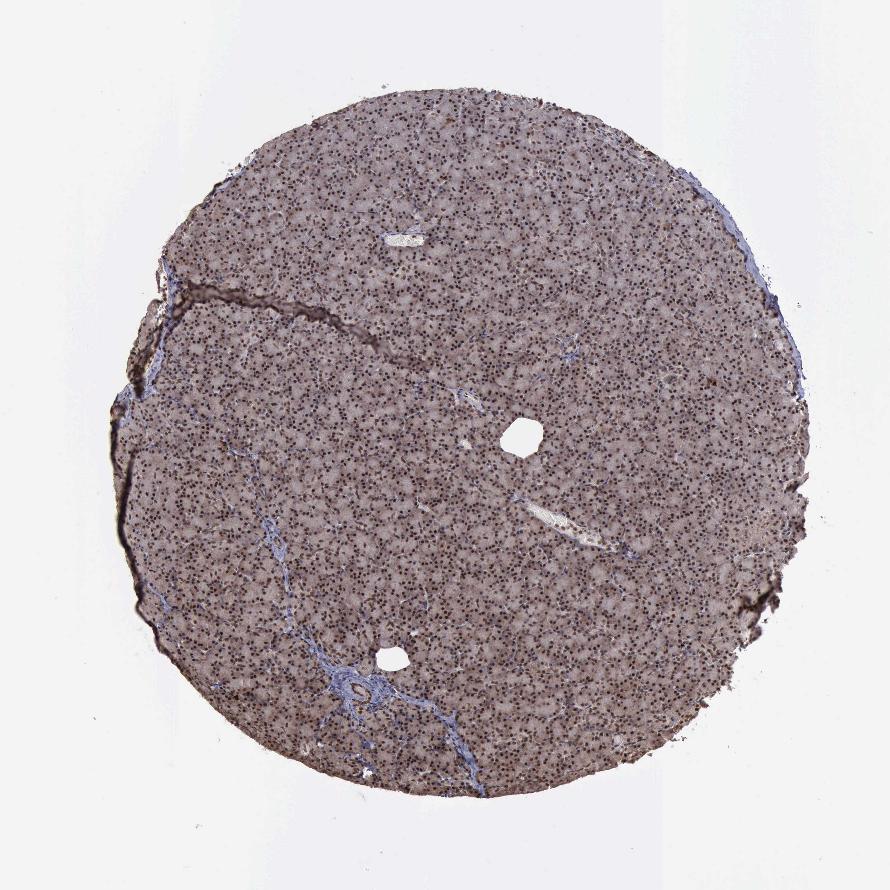

Supplement: Supplementary file 1 [file Presentation1.zip › Supplementary/Additional File 2/MT2A_Normal.jpg]

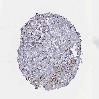

Supplement: Supplementary file 1 [file Presentation1.zip › Supplementary/Additional File 2/MT2A_Tumour.jpg]

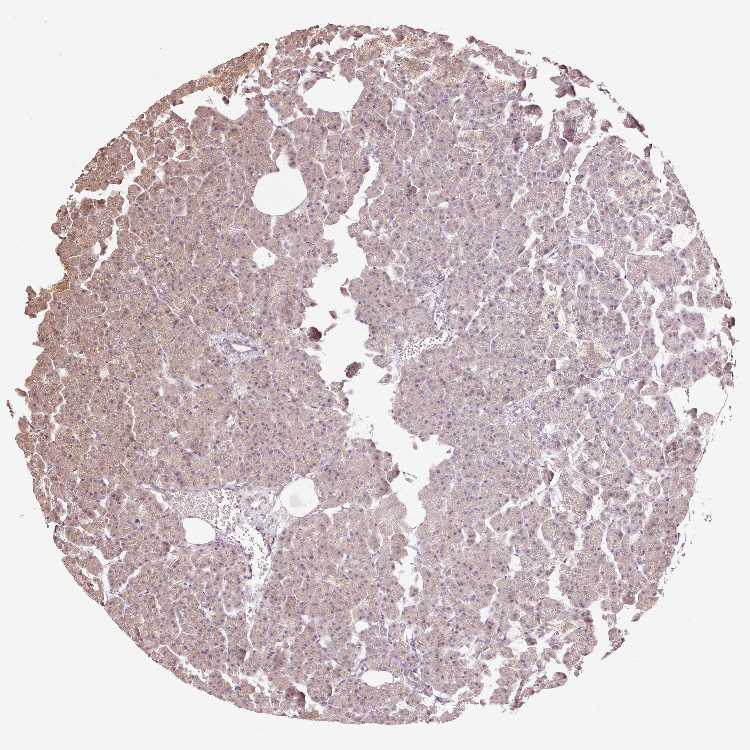

Supplement: Supplementary file 1 [file Presentation1.zip › Supplementary/Additional File 2/OXER1_Normal.jpg]

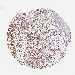

Supplement: Supplementary file 1 [file Presentation1.zip › Supplementary/Additional File 2/OXER1_Tumour.jpg]

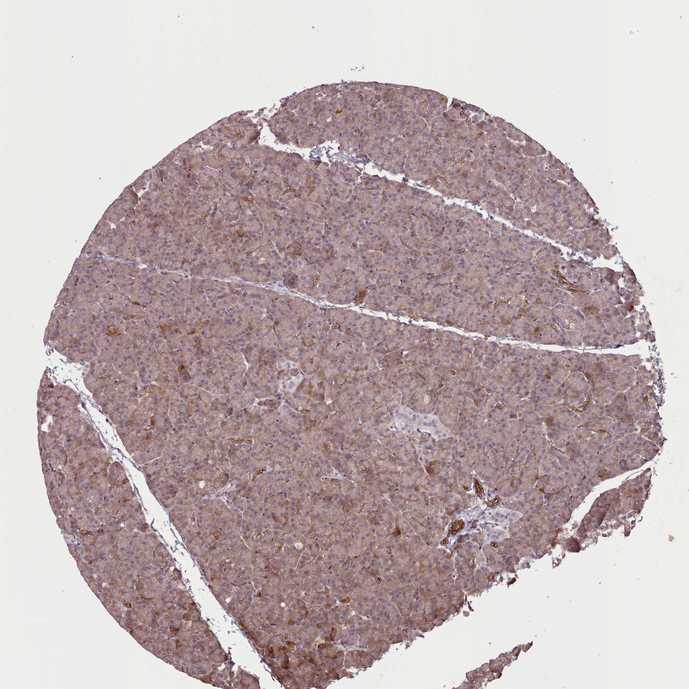

Supplement: Supplementary file 1 [file Presentation1.zip › Supplementary/Additional File 2/PLD4_Normal.jpg]

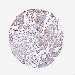

Supplement: Supplementary file 1 [file Presentation1.zip › Supplementary/Additional File 2/PLD4_Tumour.jpg]

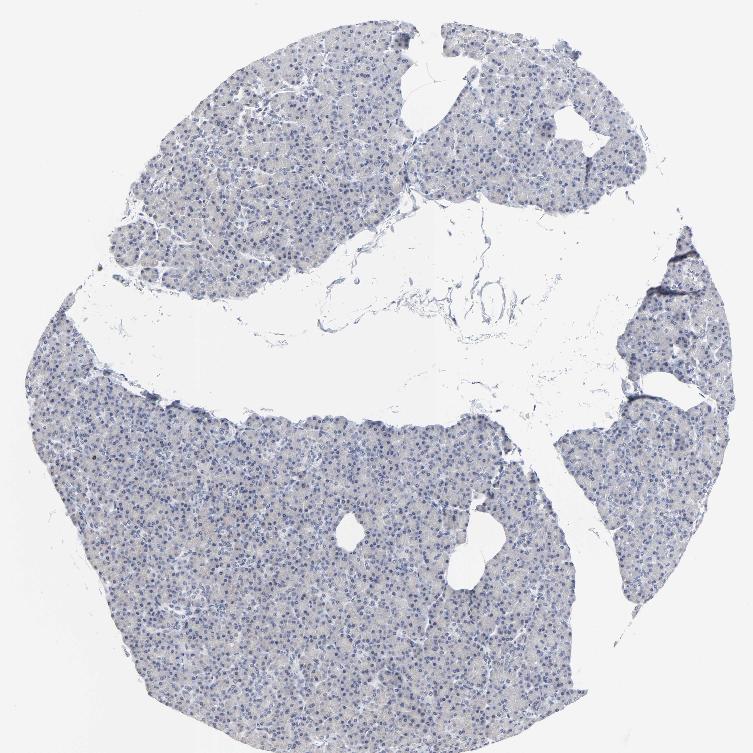

Supplement: Supplementary file 1 [file Presentation1.zip › Supplementary/Additional File 2/ZNF589_Normal.jpg]

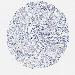

Supplement: Supplementary file 1 [file Presentation1.zip › Supplementary/Additional File 2/ZNF589_Tumour.jpg]
